# Cohesin's Concatenation of Sister DNAs Maintains Their Intertwining

**DOI:** 10.1016/j.molcel.2011.07.034

**Published:** 2011-10-07

**Authors:** Ana-Maria Farcas, Pelin Uluocak, Wolfgang Helmhart, Kim Nasmyth

**Affiliations:** 1 Department of Biochemistry, University of Oxford, Oxford OX1 3QU, UK

## Abstract

The contribution of DNA catenation to sister chromatid cohesion is unclear partly because it has never been observed directly within mitotic chromosomes. Differential sedimentation-velocity and gel electrophoresis reveal that sisters of 26 kb circular minichromosomes are held together by catenation as well as by cohesin. The finding that chemical crosslinking of cohesin's three subunit interfaces entraps sister DNAs of circular but not linear minichromosomes implies that cohesin functions using a topological principle. Importantly, cohesin holds both catenated and uncatenated DNAs together in this manner. In the vicinity of centromeres, catenanes are resolved by spindle forces, but linkages mediated directly by cohesin resist these forces even after complete decatenation. Crucially, persistence of catenation after S phase depends on cohesin. We conclude that by retarding Topo II-driven decatenation, cohesin mediates sister chromatid cohesion by an indirect mechanism as well as one involving entrapment of sister DNAs inside its tripartite ring.

## Graphical abstract

**Graphical Abstract:**

**Figure:**
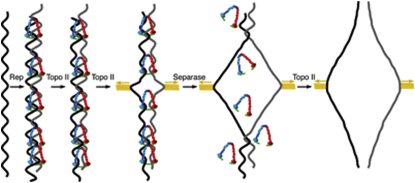

**Highlights:**

► Sister chromatid intertwining can be detected in mitotic cells ► Physical cohesion is generated through arm loci as well as centromeres ► Sister chromatid intertwining is maintained by cohesin ► Cohesin holds sister chromatids together independent of SCI

## Introduction

By resisting forces exerted by microtubules, sister chromatid cohesion (SCC) generates the tension required to stabilize kinetochore-microtubule connections and is essential for the eventual segregation of sisters to opposite poles at anaphase. Two mechanisms are capable of holding sister DNAs together: intertwining (catenation) of sister DNAs (Murray and Szostak, 1985; Sundin and Varshavsky, 1981; Surosky et al., 1986) and proteinaceous connections mediated by a multisubunit complex called cohesin (Guacci et al., 1997; Losada et al., 1998; Michaelis et al., 1997). Both are established during DNA replication, but the observation that sister DNAs of circular minichromosomes are fully decatenated by the time yeast cells enter mitosis (Koshland and Hartwell, 1987) has raised doubts as to whether sister chromatid intertwining (SCI) survives long enough to facilitate chromosome segregation.

At the heart of the cohesin complex is a tripartite ring composed of its Smc1, Smc3, and α-kleisin subunits (Scc1). Smc1 and Smc3, which are rod-shaped molecules with ABC-like ATPase domains (NBDs) at one end and a dimerization domain at the other, form V-shaped heterodimers whose ATPase heads are interconnected by Scc1 (Haering et al., 2002, 2004; Hirano and Hirano, 2002). Cohesin loads onto chromosomes during G1 with the aid of a distinct Scc2/4 complex (Ciosk et al., 2000; Furuya et al., 1998; Michaelis et al., 1997), generates cohesion during S phase through a process involving modification of its Smc3 ATPase domain by the Eco1 acetyl transferase (Rolef Ben-Shahar et al., 2008; Ivanov et al., 2002; Rowland et al., 2009; Unal et al., 2008; Zhang et al., 2008a), and is finally removed from chromosomes through cleavage of Scc1 by a thiol protease called separase (Uhlmann et al., 1999, 2000). Scc1 cleavage is not only necessary for sister chromatid disjunction at anaphase but also sufficient (Oliveira et al., 2010; Uhlmann et al., 2000). The finding that sister chromatids can be triggered to disjoin by TEV protease in metaphase cells whose α-kleisin contains TEV recognition sites implies that SCIs, even if they are in fact still prevalent at this stage of the cell cycle, are insufficient to resist spindle forces in the absence of cohesin.

The physical properties of SCC have so far been investigated only in yeast, where differential sedimentation velocity and native gel electrophoresis have been used to distinguish monomeric circular minichromosomes from dimeric versions whose 2.3 kb long monomeric (i.e., uncatenated) sister DNAs are held together by cohesin (Ivanov and Nasmyth, 2007). The fact that cohesin forms a ring whose cleavage triggers sister chromatid disjunction both in vivo and in vitro suggests that it acts as a topological device, entrapping sister DNAs inside its ring. Recent experiments have confirmed a key prediction of this hypothesis, namely that chemical crosslinking of the tripartite ring's three interfaces should be sufficient to trap sister DNAs within a structure resistant to protein denaturation (Haering et al., 2008).

To explore the physical nature of cohesion within artificial chromosomes more resembling natural ones, we have extended differential sedimentation-velocity and gel electrophoresis to 26 kb circular and 42 kb linear chromosomes. In addition to revealing centromere-dependent and independent cohesion as well as cohesion between linear chromatids, we describe cohesin-dependent SCI in mitotic cells. Importantly, crosslinking experiments confirm that a topological principle applies to the mechanism by which cohesin holds 26 kb as well as 2.3 kb minichromosomes. We conclude that cohesin holds sister DNAs together using direct and indirect mechanisms, directly by embracing sister DNAs inside its ring and indirectly by retarding removal of SCIs by Topo II.

## Results

### Detection of SCI in Mitotic Cells

Homologous recombination-mediated gap repair was used to create a small circular chromosome containing the *ARS1* origin, the *TRP1* gene, the backbone of pUC19, and 22 kb of DNA surrounding and including *CEN3* (Figure S1A). Following transfer to *E. coli*, the 26 kb minichromosome was subsequently introduced into a variety of yeast strains by DNA transformation (Table S1).

**Figure 1:**
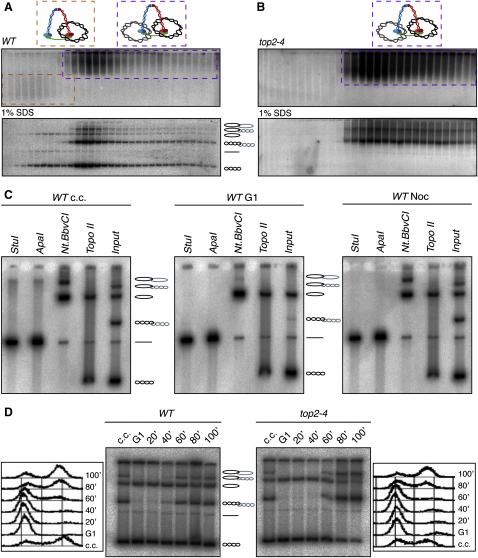
Detection of Sister Chromatin Intertwining in Mitotic Cells (A) Separation and detection by differential sedimentation and Southern blot, respectively, of monomers (dashed orange boxes) and dimers (dashed purple boxes) of large circular minichromosomes extracted from wild-type cells (K16150) grown at 25°C. Following heat denaturation in 1% SDS, the minichromosome population is resolved into six different DNA species. (B) *TOP2* protein inactivation by growth of *top2-4* strain (K17890) at 37°C converts the entire minichromosome population into catenated DNAs. (C) Identification by treatment with restriction enzymes (StuI and ApaI), nicking enzyme (*Nt.BbvCI*), and recombinant *topoisomerase II* protein (*Topo II*) of the six individual DNA species (from bottom upwards): supercoiled circle, linear monomer, supercoiled-supercoiled catenanes, nicked circle, nicked-supercoiled catenanes, and nicked-nicked catenanes. (D) Documenting the formation of DNA catenanes during a cell cycle. Wild-type (K16150) and *top2-4* (K17890) cells arrested with α factor pheromone were released at 37°C into rich media containing nocodazole. Time points were collected every 20 min for genomic DNA preparation and FACS.

Cleared lysates prepared from wild-type cells arrested in a mitotic state by addition of nocodazole (Figure S1B) were sedimented in 10%–45% sucrose gradients and minichromosome-containing fractions electrophoresed under native conditions through 0.5% agarose gels. Southern blotting revealed two fractions of minichromosome DNA: a minor “monomer” fraction that sediments slowly but electrophoreses rapidly and a more abundant “dimer” fraction that sediments rapidly but electrophoreses slowly (Figure 1A, upper panel). This assignation is confirmed by the absence of monomers from *top2-4* cells that have undergone S phase at the restrictive temperature, where the products of replication are known to be catenated dimers (Figure 1B). Importantly, the sedimentation velocity of the dimeric form in wild-type cells was similar if not identical to that of minichromosomes from *top2-4* cells (Figures 1A, 1B, 1D, and S1C). Aggregation of some minichromosomes during centrifugation may be responsible for their faster-than-expected sedimentation velocities, i.e., peaks trailing toward the bottom of the gradients.

Electrophoresis following denaturation of sucrose gradient fractions by heating to 65°C in 1% SDS revealed that some minichromosomes migrated with a velocity expected of catenated sister DNA dimers while others migrated with a velocity expected of monomeric DNAs (Figure 1A, lower panel). Importantly, what we presume to be catenated DNA dimers were only present in “dimer” sucrose gradient fractions. To confirm the identity of the various electrophoretic forms observed after SDS treatment, we pretreated DNA samples isolated from cycling, G1- arrested, or nocodazol-arrested cells either with restriction enzymes, with the *Nt.BbvCI* nicking enzyme, or with Topo II (Figure 1C). Restriction with enzymes having a unique site within the minichromosome DNA (StuI and ApaI) converted all DNAs to a monomeric linear form, while *Nt.BbvCI* converted supercoiled species to more slowly electrophoresing nicked circles. In the case of DNA species from cycling or nocodazole-arrested cells, *Nt.BbvCI* produced two types of relaxed circles; the majority were monomeric while a minority (∼30% in the experiment shown) were dimeric and presumably catenated nicked circle dimers.

Crucially, Topo II converted all forms presumed to be catenated dimers to either supercoiled or nicked monomers. Moreover, catenated dimers were absent from cells arrested in G1 by α factor but appeared upon their release as cells underwent DNA replication (Figures 1D and S1C). Lastly, replication under conditions where Topo II activity is lowered, namely *topo2-4* cells at 37°C, caused most DNAs to accumulate in this form (Figures 1B, 1D, and S1C). Catenated dimers of circular minichromosomes have never previously been detected at appreciable levels in wild-type cells (Koshland and Hartwell, 1987). They are largely absent from dimeric fractions of 2.3 kb minichromosomes (Ivanov and Nasmyth, 2007) and infrequent in dimeric fractions of 4.5 and 7.5 kb minichromosomes (Figure S2). We suggest that their incidence is proportional to minichromosome size (Figure S2).

### Chromosome Arms as well as Centromeres Generate Physical Cohesion

**Figure 2:**
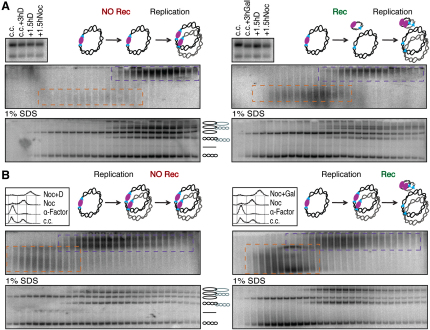
Chromosome Arms as well as Centromeres Generate Physical Cohesion (A) Asynchronous K18066 cultures having the *Z. rouxii* recombinase under the control of the Gal1 promoter (*Gal1P-Rec*) and a 26 kb circular minichromosome containing *RS-Cen3-RS* (whose centromere can be uncoupled upon recombinase expression) were grown at 30°C in YEPraff; glucose (Cen3 present) or galactose (Cen3 deleted) were added to 2% final concentration for 3 hr. Cells were shifted into YPD for 1.5 hr, followed by nocodazole arrest for 1 hr 40 min. (B) K18066 cultures having the 26 kb circular minichromosome bearing the *RS-Cen3-RS* were arrested at 30°C with the α factor pheromone in YEPraff and released for 1 hr in nocodazole. Glucose or galactose were added to 2% final concentration and incubated for an additional 3 hr, maintaining the nocodazole arrest.

Cohesion associated with 2.3 kb minichromosomes is abolished by removal of their centromeres, implying that the cohesion established on such minute chromosomes is established and maintained exclusively by their kinetochores (Haering et al., 2008). The creation of much larger minichromosomes whose cohesion can still be measured by physical means provides an opportunity to address whether cohesion is also established by pericentric sequences. To test whether 26 kb minichromosomes can establish cohesion without their centromeres, we used gap-mediated homologous recombination in strain PMY185 (Megee et al., 1999) to create a version of the minichromosome whose *CEN3* sequence is flanked by recombination sites recognized by the *Z. rouxii* recombinase (*RS-Cen3-RS*). Cells with the recombinase under control of a galactose-inducible promoter (*GalP-Rec*) were grown to exponential phase in YEP raffinose (YEPraff) medium, and the culture was split. One half was transferred to YEP glucose (YEPD) and the other to YEP galactose (YEPgal). After 3 hr, by which time recombinase induction had caused complete deletion of *CEN3* in YEPgal but not in YEPD (Figure 2A), both cultures were transferred to fresh YEPD and incubated for one generation time (1.5 hr) before arresting them in mitosis by growth in the presence of nocodazole for a further 1.5 hr. Analysis of sucrose gradient fractions from the two cultures revealed that the fraction of minichromosomes in dimer fractions was reduced about 2-fold in the cells transiently exposed to galactose medium and therefore lacking *CEN3* for one or two rounds of DNA replication prior to their mitotic arrest (Figure 2A). This implies that about half of the cohesion established on 26 kb minichromosomes is mediated by centromeres while the other half is mediated by pericentric sequences. Importantly, greater than 50% of the DNA molecules lacking *CEN3* from dimer fractions were monomeric, and the dimeric nature of these can therefore be attributed exclusively to cohesin.

To assess the contribution of centromere and arm sequences to the maintenance of cohesion, we analyzed the effect of deleting *CEN3* in nocodazole-arrested cells. Cells growing in YEPraff arrested in G1 by α factor were released into medium containing nocodazole. After replication and mitotic arrest, the culture was split and incubated for a further 3 hr in the presence of nocodazole either in YEPD (*CEN3* present) or in YEPgal (*CEN3* deleted). Gradients prepared from the two cultures revealed that *CEN* deletion reduced (approximately 2-fold) the fraction of dimeric minichromosomes (Figure 2B). It also appeared to increase modestly the fraction of dimers that were catenated, an effect that in this case could be caused by the recombination process. We conclude that a sizeable fraction of the cohesion associated with 26 kb minichromosomes depends on the continued presence of centromere sequences, raising the possibility that cohesin complexes might have a higher affinity for this chromatin address. The experiments also imply that cohesion established by centromeres does not spread into arm sequences in a manner that can subsequently resist centromere removal.

### Measuring Sister Chromatid Cohesion of Linear Minichromosomes

Our physical assay has hitherto been confined to measuring cohesion associated with circular minichromosomes. As predicted by the ring model, linearization with a restriction enzyme in vitro causes cohesin to dissociate from small circular minichromosomes (Ivanov and Nasmyth, 2005). It also causes loss of SCC (Ivanov and Nasmyth, 2007). How then does cohesin hold the chromatids of natural chromosomes together? One possibility is that these contain obstacles to cohesin's diffusion along chromatin fibers. Sites of convergent transcription, large transcription complexes and/or factories, and telomere caps are all possible candidates. Interesting in this context is the observation that short linear artificial chromosomes missegregate at high frequencies (Surosky et al., 1986), a phenomenon that might be due to a failure to retain sufficient cohesion.

**Figure 3:**
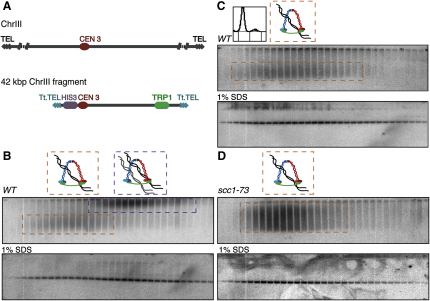
Measuring Sister Chromatid Cohesion of Linear Minichromosomes (A) Schematic representation of the 42 kb linear minichromosome (modified from Murray et al., 1986, with permission from Elsevier). (B) Differential sedimentation and Southern blot resolution of monomers (dashed orange boxes) and dimers (dashed purple boxes) of linear minichromosomes extracted from wild-type cells (K18251) arrested in metaphase. (C) Linear minichromosome profiles obtained from G1-arrested cells (K18251) contain exclusively monomeric DNA species. (D) *SCC1*'s inactivation by cellular growth of *scc1-73* strain (K18249) for 2 hr at 37°C in the presence of nocodazole causes the loss of linear minichromosome dimers.

To investigate whether it is possible to detect cohesion between linear chromatids in vitro using differential sedimentation velocity/electrophoresis, we analyzed a 42 kb acrocentric minichromosome that is lost at a rate of 10^−2^ per mitotic division (Murray et al., 1986). Its DNA contains a centromere-containing fragment of chromosome III, artificial telomeric sequences from the *Tetrahymena* rDNA, the auxotrophic markers *HIS3* and *TRP1*, and the *ARS1* replication origin (Figure 3A) (Murray et al., 1986). Cleared lysates prepared from wild-type cells arrested in mitosis by nocodazole were sedimented in 10%–45% sucrose gradients, fractions subsequently resolved on 0.4% agarose gels, and DNAs detected by Southern blotting (Figure 3B). As in the case of circular chromosomes, this revealed two discrete populations: one slow-sedimenting and rapidly electrophoresing and another fast-sedimenting and slowly electrophoresing. Cells arrested in G1 by α factor contained the former but not the latter (Figure 3C), and we therefore presume that these represent monomers and dimers, respectively. Crucially, the absence of dimers from *scc1-73* cells incubated at the restrictive temperature implies that they are held together by cohesin (Figure 3D). Electrophoresis of gradient fractions denatured by SDS showed that all DNA molecules from monomer and dimer fractions were linear monomers. Our experiments do not distinguish whether the intertwining of these small linear chromatids is lost in vivo and/or in vitro.

### Chemical Circularization of Cohesin Entraps Circular but Not Linear Sister Minichromosome DNAs

The sister DNAs of 2.3 kb circular minichromosomes are held together by cohesin in a topological embrace—that is, they are chemically entrapped by crosslinking the tripartite ring's three interfaces (Haering et al., 2008). To test whether this is also an attribute of cohesin holding the larger, more natural chromosomes described above, we introduced the 26 kb circular or 42 kb linear minichromosomes into yeast strains containing cohesin complexes in which one, two, or all three interfaces can be crosslinked. This was performed either by fusing two genes, in the case of the Smc3/Scc1 interface, or by a thiol-specific bifunctional crosslinker, dibromobimane (bBBr), which under oxidizing conditions joins reduced cysteine pairs placed at Smc1/Smc3 and Smc1/Scc1 interfaces.

**Figure 4:**
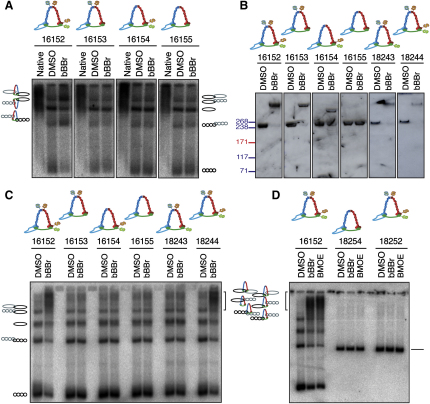
Chemical Circularization of Cohesin Entraps Circular but Not Linear Sister Minichromosome DNAs (A–C) Dimer fractions held together by modified cohesin complexes that allow the covalent closure of one, two, or all three cohesin interfaces were treated with DMSO or bBBr, denatured with 1% SDS, and separated in 0.45% agarose gel (A), 4%–8% Tris-acetate gels (B), or 0.45% agarose gel containing 0.2% SDS (C). Southern blots were hybridized with α^32^P-labeled probe (A and C); Smc3-TEVx3-Scc1-HA6 protein was detected on western blots with α-HA antibody (B). (D) Dimers of 26 kb circular or 42 kb linear minichromosomes held together by modified cohesin complexes were treated with DMSO, bBBr, or BMOE, denatured in 1% SDS, separated in 0.45% agarose gel containing 0.2% SDS, and detected by Southern blotting.

Cleared lysates were sedimented through 10%–45% sucrose gradients and fractions containing monomeric and dimeric minichromosomes detected by Southern blotting following agarose gel electrophoresis (Figure S3A). Dimer fractions were dialyzed to remove DTT, sucrose, and other low-molecular-weight contaminants and treated either with the solvent DMSO or with bBBr (Haering et al., 2008). After quenching the reactions with DTT, samples were heat denatured in the presence of 1% SDS and separated in 0.45% agarose gels, and minichromosomal DNA was detected by Southern blotting. bBBr induced major changes in the electrophoretic mobility of 26 kb circular DNAs from dimer fractions isolated from cells with cysteine pairs at both Smc1/Smc3 and Smc1/Scc1 interfaces, but not from cells with cysteine pairs at only one or at neither of these (Figure 4A). DNAs comigrating with supercoiled-supercoiled, supercoiled-nicked, and nicked-nicked catenanes were increased, while monomeric DNAs, both supercoiled and nicked circles, were decreased. Due to their dependence on cohesin's circularization, these changes must arise due to entrapment of DNAs inside cohesin rings.

Unlike equivalent experiments with 2.3 kb minichromosomes (Haering et al., 2008), the present experiment did not reveal new bands corresponding to DNAs concatenated by cohesin since, due to the greatly increased chromosome size, these comigrated with pre-existing DNA-mediated catenanes. It was also impossible for the same reason to determine whether cohesin had created additional catenation within DNA-mediated catenanes. To avoid this problem, we repeated the experiment, this time including SDS (0.2%) in the agarose gels. Our rationale was that SDS causes denaturated polypeptides to adopt a highly extended conformation (Gabriel and Zentner, 2005; Shinagawa et al., 1979), which might alter the electrophoretic properties of protein- but not DNA-mediated catenanes. Analysis of samples by SDS-PAGE and western blotting confirmed that bBBr induced efficient crosslinking of the various cysteine pairs; note that the cohesin proteins detected in the gradient fractions are not exclusively or even mainly those associated with minichromosome DNA (Figure 4B).

Under these circumstances, bBBr not only reduced the abundance of all existing bands but also caused the appearance of molecules with a greatly reduced mobility at the top of the gels (Figure 4C), an effect that only occurred if both Smc1/Smc3 and Smc1/Scc1 interfaces contained the appropriate cysteine pairs. Because this effect was abolished by the absence of just one of the four cysteine substitutions (compare strains 18243 and 18244), the slowly migrating DNAs at the top of the gel must be versions of all five major species of DNA, namely supercoiled and nicked monomers and all three types of dimeric DNA catenanes, that have been entrapped by circularized cohesin rings. These data imply that cohesin creates topological linkages not only between monomeric sister DNAs but also between DNAs that contain SCI. The fraction of DNA molecules whose mobility was shifted by cohesin's circularization was high, between two thirds and three quarters. Since the efficiency of cohesin circularization is probably less than 50% (Haering et al., 2008), these numbers suggest that at least one and possibly more cohesin rings concatenate each native dimer chromosome in our gradient fractions. Because the frequency of chemical entrapment is about twice that observed with 2.3 kb minichromosomes, which unlike the 26 kb version cannot form arm cohesion, these numbers suggest that cohesion mediated by chromosome arms also involves entrapment of DNAs inside cohesin rings.

Importantly, chemical circularization of cohesin in dimer fractions of 42 kb linear chromosomes caused no change in DNA mobility, with all molecules running as linear monomers (Figure 4D). We presume that the cohesin rings slide off linear DNAs upon SDS-mediated protein denaturation, at least during the extensive period of electrophoresis. This result is an important confirmation of the topological nature of cohesin's linkage of sister DNAs. It also emphasizes that native cohesin rings on native chromosomes are less free to diffuse along chromatin fibers than denatured rings along naked DNA.

### Maintenance but Not Establishment of SCI Depends on Cohesin

**Figure 5:**
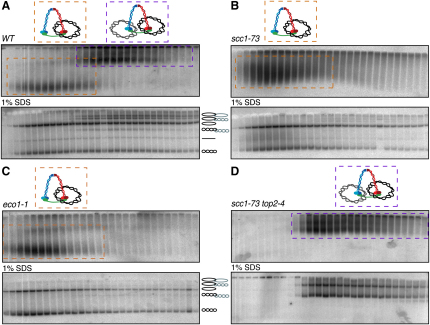
Maintenance but Not Establishment of SCI Depends on Cohesion (A) Wild-type or mutant strains were arrested with α factor pheromone and released into rich media containing nocodazole for 1.5 hr at 37°C. Separation of monomeric and dimeric large circular minichromosomes extracted from wild-type cells (K16150) is shown (A). *SCC1*'s inactivation due to growth of *scc1-73* strain (K17889) at restrictive temperature causes complete depletion of dimeric minichromosome species (B). *ECO1* inactivation by growth of *eco1-1* strain (K17888) at restrictive temperature depletes dimeric minichromosomes (C). Concomitant inactivation of *SCC1* and *TOP2* proteins by growing the *scc1-73 top2-4* double mutant strain (K17892) at restrictive temperature mimics the inactivation of *TOP2* protein alone, ensuing total lack of monomeric DNAs (D).

**Figure 6:**
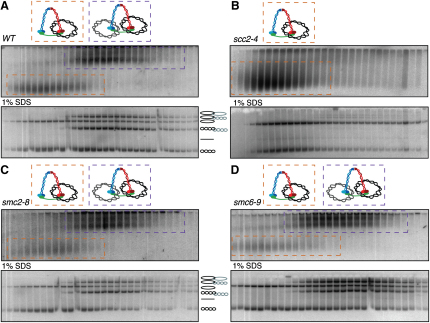
SCI Is Largely Unaffected by Condensin and the Smc5/6 Complex (A) Wild-type or mutant strains were arrested with α factor pheromone and released into rich media containing nocodazole for 1.5 hr at 37°C. Separation of monomeric and dimeric large circular minichromosomes extracted from wild-type cells (K16150) is shown in (A). (B) *SCC2*'s inactivation due to growth of *scc2-4* strain (K16149) at restrictive temperature causes complete depletion of dimeric minichromosome species. (C and D) Separation of monomeric and dimeric large circular minichromosomes extracted from *smc2-8* (K17893) or *smc6-9* (K17895) strains grown at restrictive temperature to induce the inactivation of the *SMC2* (C) or *SMC6* (D) proteins, respectively.

The finding that a substantial fraction (30%–50%) of circular 26 kb dimers are intertwined raises the possibility that this population of dimers might be independent of cohesin. In fact, our crosslinking experiments (Figure 4C) suggest that cohesin entraps catenated as well as uncatenated sister DNAs together. To address whether catenation can hold sisters together in cohesin's absence, we measured the effect of a temperature-sensitive mutation (*scc1-73*) that disrupts binding of the C-terminal domain of α-kleisin to Smc1's NBD (Haering et al., 2004). Gradients prepared from synchronous cultures of wild-type and mutant cells revealed that native dimers were abolished by the cohesin mutation when cells replicated and entered a nocodazole-induced mitotic arrest at the restrictive temperature (Figure 5B, upper panel). This implies that SCI in mitotic cells depends on cohesin, a conclusion confirmed by electrophoresis of gradient fractions after SDS treatment, which revealed few if any catenated dimers (Figure 5B, lower panel). Equivalent experiments with *scc2-4* and *eco1-1* (G211D) mutant cells showed that catenated dimers were equally dependent on cohesin's Scc2/4 loading complex and on the Eco1 acetyl transferase (Figures 6B and 5C). The latter result implies that mere association of cohesin with chromosomal DNA is insufficient. SCI persistence requires that cohesin actually forms cohesion. To address whether cohesin is required to form SCI in the first place or merely to maintain some SCI after S phase is completed, we analyzed *scc1-73 top2-4* double mutant cells after a single round of replication at the restrictive temperature. This revealed that all DNAs accumulated as catenated dimers (Figure 5D), implying that cohesin is required to maintain SCI but not to form it in the first place. This result also implies that decatenation by Topo II is responsible for the loss of SCI in cohesin mutants.

### SCI Is Largely Unaffected by Condensin and the Smc5/6 Complex

Detection of SCI in wild-type cells enabled us to address the roles of Smc5/6 complexes in stabilizing SCI behind replication forks (Kegel et al., 2011) and condensin in facilitating Topo II-mediated resolution (Coelho et al., 2003; D'Ambrosio et al., 2008). Condensin (*smc2-8* and *ycg1-10*) and Smc5/6 mutations (*smc6-9*) might be expected to increase and decrease the frequency of catenated dimers, respectively. Unlike cohesin mutations, neither *smc2-8* and *ycg1-10* nor *smc6-9* mutations had any obvious effect on formation of dimeric minichromosomes following replication at the restrictive temperature in the presence of nocodazole (Figures 6C, 6D, S4A, and S4B). Importantly, these mutations also had little or no effect on the incidence of catenation among dimeric minichromomes, a result confirmed by analysis of DNAs at different time points after release from α factor (Figure S4B).

### Cohesin Holds Sisters Together Independent of SCI

**Figure 7:**
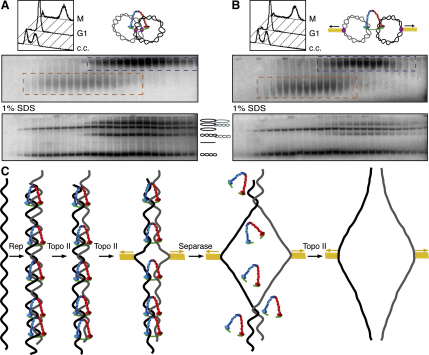
Cohesin Holds Sister DNAs Together Independent of SCIs (A and B) Yeast strains (K18072) having *Cdc20* under the control of the repressible *Met3* promoter (*Met3P-Cdc20*) and bearing the 26 kb circular minichromosome were arrested with α factor pheromone in −Met-TrpRaff medium and released into YPD + 200 mM methionine with or without nocodazole. Minichromosome cohesion was assessed in the presence (B) or absence (A) of microtubule forces on sister kinetochores. (C) DNA catenation throughout the cell cycle. During DNA replication (Rep), SCIs arise concurrently with cohesion establishment. Topo II-driven decatenation takes place throughout S and G2 phases, but complete decatenation is hampered by cohesin's embrace of sister DNAs. Following separase-mediated cohesin removal, Topo II resolves the residual DNA catenanes ensuing complete sister separation.

Due to the presence of nocodazole in the experiments described above, cells entered a mitotic state in the absence of microtubules. To assess the effect of spindle forces, we used α factor to arrest in G1 cells whose APC/C Cdc20 activator protein is expressed from the methionine-repressible *MET3* promoter and then transferred them into rich medium lacking pheromone but containing methionine in the presence and absence of nocodazole. In the absence of the drug, cells replicate their chromosomes and subsequently arrest in metaphase due to Cdc20 depletion, which prevents separase activation and mitotic exit. Analysis of gradients prepared from cells incubated with and without nocodazole revealed that spindle forces reduced, albeit modestly, the fraction of dimeric minichromosomes (Figures 7A and 7B). Possibly more significant, they caused a major reduction in the fraction of native dimers containing catenated DNAs. Thus, in cells where the minichromosomes are subject to spindle forces, most dimers were composed of monomeric DNAs held together solely by cohesin (Figure 7B). This has two important implications. First, spindle forces facilitate decatenation even when sister chromatids remain held together by cohesin (Figure 7C). If, as seems likely, spindle forces facilitate decatenation by subjecting SCI to tension, then our observations imply that cohesin does not interfere with this process. Given the size of cohesin's tripartite ring, biorientation of circular minichromosomes would bring forces to bear on SCIs before they would do so on DNAs entrapped inside cohesin rings. The second implication is that cohesin does not require SCI to resist spindle forces. Thus, cohesin can resist spindle forces without SCI, but SCI cannot do so without cohesin (Oliveira et al., 2010; Uhlmann et al., 2000).

## Discussion

Cohesin is a key constituent of both interphase and mitotic chromosomes. It not only mediates SCC necessary for chromosome segregation and DNA repair, but also, at least in animal cells, regulates gene expression during embryonic development. Understanding how cohesin interacts with DNA is essential if we are to grasp how it mediates such diverse functions. Previous experiments have described the use of differential sedimentation velocity and gel electrophoresis to measure and probe the physical properties of cohesin-mediated linkages between very small (2.3 kb) circular sister minichromosomes. The main conclusion from such studies, namely that cohesin entraps sister DNAs inside its tripartite ring structure, has been questioned (Heidinger-Pauli et al., 2010; Skibbens, 2010; Yanagida, 2009; Zhang et al., 2008b) partly on the grounds that such small minichromosomes are dominated by their centromeres and may have unique properties due to their circularity.

We show here that the same techniques can be applied to a 26 kb circular chromosome, which unlike the 2.3 kb version contains extensive arm sequences responsible for about half of the SCC. Unlike smaller minichromosomes, mitotic chromosomes of this size (in nocodazole-arrested cells) retain some of the SCI produced during DNA replication, demonstrating that SCI as well as cohesin links sister chromatids together during mitosis. If the incidence of DNA catenanes within 26 kb circles is similar to that of SCI within natural linear chromosomes, then SCI may occur every 50–100 kb within real chromosomes.

What is the interdependence of these two types of cohesion? Our observation that a large fraction of dimeric 26 kb minichromosomes are composed of monomeric, i.e., uncatenated DNAs implies that cohesion mediated by cohesin does not require persistent SCI. Indeed, the finding that chromatids remain cohesed after biorientation on metaphase spindles despite almost complete decatenation (Figure 7B) proves that linkages mediated directly by cohesin counteract microtubule-driven sister chromatid disjunction in vivo in the absence of SCI as well as merely hold sisters together in vitro. In contrast, most SCI associated with mitotic 26 kb minichromosomes is abolished by mutations affecting cohesin. Thus, SCI depends on direct cohesin linkages but not vice versa. By holding sisters together in a manner independent of SCI, cohesin presumably prevents complete decatenation by Topo II until cohesin cleavage at the metaphase to anaphase transition (Figure 7C). It is nevertheless important to stress that our experiments do not exclude a role of SCI in the initial establishment of cohesion during the process of DNA replication. To address possible functions of SCI, it will be necessary to find mutations that affect its formation. It was partly for this reason that we tested the effect of mutations inactivating Smc5/6 complexes, which have been proposed to stabilize SCI behind replication forks (Kegel et al., 2011). Our finding that the *smc6-9* mutation had little or no effect is difficult to reconcile with the above proposal.

Our observation that spindle forces reduce minichromosome catenation demonstrates something that has long been suspected, namely that tension can drive SCI resolution. The observation that mitotic spindles induce a massive topological change to minichromosomes whose sisters are multiply intertwined due to a lack of Topo II activity has led to the speculation that an equivalent process in wild-type cells induces a wave of supercoiling that facilitates decatenation by Topo II (Baxter et al., 2011). Though our findings are not inconsistent with this proposal, in our view a more prosaic and indeed simpler explanation is that spindle forces merely bring SCIs under the sort of tension that facilitates their resolution by Topo II despite the presence of direct cohesin linkages. Besides, a putative wave of supercoiling brought about by spindle forces cannot be necessary to drive decatenation reactions, as we observe very extensive if not complete SCI resolution in cells lacking microtubules due to the presence of nocodazole when cohesin is inactivated. Consistent with the notion that loss of cohesin can trigger extensive decatenation in the absence of spindle forces is the observation that chromosome arms in animal cells fully disjoin in cells arrested in a mitotic state by spindle poisons, an event triggered by the complete removal of cohesin from chromosome arms by the prophase pathway (Waizenegger et al., 2000).

Though SCI provides a second type of linkage dependent on cohesin, it cannot alone resist spindle forces, as artificial cleavage of cohesin rings in metaphase-arrested cells is sufficient to induce complete sister chromatid disjunction (Oliveira et al., 2010; Uhlmann et al., 2000). We suggest that under these circumstances, cohesin cleavage removes the sole obstacle to full decatenation by Topo II, a notion consistent with the finding that the disjunction of sister chromatids induced by treatment of mitotic chromosomes with separase in vitro depends on Topo II activity (Wang et al., 2010). We envision two potential mechanisms by which cohesin inhibits SCI resolution. If cohesin were located at SCI loci, then it could in principle directly regulate (in this case inhibit) Topo II activity, thereby preventing complete decatenation. It is interesting in this regard that the Smc-like MukBEF complex is thought to have the opposite effect in *E. coli*, namely to facilitate decatenation of sister DNAs, by directly binding to Topo IV (Hayama and Marians, 2010; Li et al., 2010). Alternatively, the proximity of sister DNAs brought about by their concatenation by cohesin rings might facilitate the reverse reaction, namely catalysis of sister DNA catenation by Topo II.

We find that crosslinking the three interfaces of cohesin's Smc1-Smc3-Scc1 tripartite ring entraps a large proportion of sister DNAs of dimeric 26 kb minichromosomes inside circularized cohesin rings. We suggest that the reason not all DNAs are entrapped is because circularization efficiency is less than 50% (Haering et al., 2008). Because catenated as well as uncatenated DNAs are entrapped, linkages mediated directly by cohesin must coexist with SCIs. Importantly, we observed no entrapment of dimeric forms of 42 kb linear minichromosomes, an important finding confirming the topological nature of cohesion mediated by cohesin. Because half the cohesion associated with 26 kb minichromosomes is mediated by their arm sequences, and because the efficiency of sister DNA entrapment is twice that of 2.3 kb sister DNAs whose cohesion is totally centromere dependent, our data suggest that arm-mediated as well as centromere-mediated cohesion involves entrapment inside cohesin rings. It should nevertheless be pointed out that the current experiments still suffer from the limitation that they use a potentially artifactual crosslink for the Smc3-Scc1 interface, namely fusion of Smc3's C terminus to Scc1's N terminus. This is due to the absence of a crystal structure for the Smc3-Scc1 interface, which remains a key challenge for the cohesin field.

Lastly, our finding that the sister chromatids of 42 kb linear minichromosomes remain linked by cohesin following sedimentation through sucrose gradients and electrophoresis for more than 48 hr through agarose gels demonstrates that chromatin fibers or structures associated with telomeres block cohesin's translocation off the ends of chromosomes. It also emphasizes that the minichromosome cohesion hitherto measured by physical means is not an artifact of their circularity. Our observations should encourage efforts to visualize individual cohesin complexes sliding along linear chromatin fibers in vitro, which promises to shed further insight into its manner of association.

## Experimental Procedures

### Plasmid Construction

Within the MCS of Yiplac204 (*TRP1*, *ARS1*, pUC19), we inserted between SbfI-BamHI and KpnI-EcoRI sites ∼3 kb DNA fragments homologous to pericentromeric regions situated ∼11 kbp leftward and rightward from *CEN3*, respectively. The XmaI linear product was repaired in vivo by homologous recombination in wild-type (K699) or in PMY185 (Megee et al., 1999), creating 26 kb minichromosomes that include a 23 kb genomic DNA fragment (ChIII: 103,000–126,169) bearing either the wild-type *CEN3* (pK6040) or the *RS-CEN3-RS* (containing recombination sites for *Z. rouxii* recombinase) (pK6039).

### Media and Growth Conditions

#### General Growth Conditions

Yeast strains bearing the 26 kb circular or 42 kb linear chromosomes were grown in synthetic media lacking tryptophan supplemented with 2% Raffinose (−TrpRaff) at 25°C. Exponentially grown cultures (OD_600_ = 0.6–0.8) were diluted to OD_600_ = 0.2 into YEPraff media and grown for 5 hr at 25°C. Cells were arrested at metaphase in the presence of 10 μg/ml nocodazole for 1 hr 45 min.

#### Protein Inactivation

Strains bearing temperature-sensitive alleles encoding various chromosome structure determinants (*scc1-73*, *scc2-4*, *top2-4*, *scc1-73 top2-4*, *smc2-8*, *eco1-1*, *smc6-9*) and wild-type-bearing minichromosomes were grown over night at 23°C in −TrpRaff medium. Exponentially growing cultures were diluted into YEPraff media to OD_600_ = 0.075, grown at 23°C to OD_600_ = 0.15 and arrested with 4 μg/ml α factor mating pheromone for 2 hr; cultures were shifted for 30 min at 37°C with additional 1 μg/ml α factor. Cells were released in YEPraff + 10 μg/ml nocodazole for 1.5 hr at 37°C.

Yeast strains containing the 42 kb linear chromosome were grown in YEPraff to OD_600_ = 0.6 at 23°C. Nocodazole-induced arrest was initiated simultaneously with the temperature shift to 37°C for 2 hr.

#### Centromere Deletion

Exponentially growing cultures of K18066 (*GalP-ZrouxiiRec::Leu2*, 26 kb:*RS-cen3-RS*) were diluted from −TrpRaff into YEPD or YEPgal to OD_600_ = 0.2 and grown for 3 hr at 30°C. Cells were shifted into YEPD for 1.5 hr, followed by nocodazole-induced arrest for 1.5 hr at 30°C.

Exponentially growing K18066 diluted into YEPraff were grown to OD_600_ = 0.6 at 30°C and incubated with 10 μg/ml nocodazole for 1 hr followed by addition of glucose or galactose to 2% for 3 hr, continuing the nocodazole arrest.

#### Mitotic Arrest by Cdc20 Depletion

Yeast strains K18072 (*MetP-Cdc20*) containing the 26 kb minichromosome grown in −Met-TrpRaff at 30°C were diluted to OD_600_ = 0.075 grown to OD_600_ = 0.15. G1 arrest was ensued with 5 μg/ml α factor for 2 hr. Cells were released into YEPraff + 2 mM methionine with or without 10 μg/ml nocodazole for 1.5 hr at 30°C.

### FACS Analysis

Approximately 0.5 × 10^7^ cells were sedimented at 2300 rcf for 1 min, and pellets were stored in 1 ml 70% ethanol at −20°C. Cells were taken in 1 ml 50 mM Tris-HCl (pH 7.5) + 20 μl of 10 mg/ml RNaseA and incubated with shaking for 2 hr at 37°C. Cells were taken in 500 μl buffer (200 mM Tris-HCl [pH 7.5], 211 mM NaCl, 78 mM MgCl_2_) and propidium iodide was added at 50 μg/ml final concentration. Samples were sonicated for 5 s at 40% power and 50–100 μl was taken into 1 ml 50 mM Tris-HCl (pH 7.5) and read with a Becton Dickinson FACSCalibur, ensuring 10,000 events per sample.

### Genomic DNA Preparation

We washed 7.5–10 ODs of cells fixed in 50% ethanol (1:1 v/v) and 10 mM EDTA with 1 ml H_2_O and transferred to 1.5 ml tubes. The cell wall was digested in 200 μl SCE buffer (1 M sorbitol, 0.1 M sodium citrate [pH 7.0], 60 mM EDTA) + 0.1 M β-mercaptoethanol + 1 mg/ml zymolyase 100T for 1 hr at 37°C. Cells were lysed in 200 μl Sol2 (2% SDS, 0.1 M Tris-HCl [pH 9.0], 50 mM EDTA) for 5 min at 65°C. Potassium acetate (200 μl) was added and samples were incubated for 20 min on ice. Cell debris were sedimented at 16,000 rcf for 10 min. Supernatant (450 μl) was precipitated with 1 ml ice-cold isopropanol + 200 μl 5 M ammonium acetate. DNA was sedimented at 2300 rcf, excess liquid was removed, and the pellet was dissolved in 90 μl TE buffer for 30 min at 37°C. DNA was reprecipitated with 10 μl 5 M ammonium acetate and 200 μl isopropanol. DNA pellet was washed with 70% ethanol and dissolved in 50 μl TE containing 0.2 mg/ml RNaseA.

### Differential Sedimentation of Cleared Lysates

Cells were harvested at 3500 rpm in Heraeus Multifuge, and pellets were washed twice with cold H_2_O, resuspended in 100 mM Tris HCl (pH 9.4), 10 mM DTT, and 10 μg/ml nocodazole, and incubated for 20 min on ice. Cells were washed with ice-cold H_2_O, resuspended in spheroplasting buffer (1 M sorbitol, 50 mM Tris HCl [pH 7.5], 1 mM CaCl_2_, 1 mM MgCl_2_, 10 μg/ml nocodazole, 350 U lyticase L4025-Sigma) and incubated 30 min on an orbital platform at 4°C. Spheroplasts were sedimented in a Beckman Coulter JA25.50 at 6000 rpm for 6 min, gently washed with 1 M sorbitol, transferred to 1.5 ml tubes, and sedimented for 1 min at 1500 rcf and 4°C. Pellets were resuspended in 200 μl cold 0.4 M sorbitol and lysed on ice for 30 min by the addition of 700 μl lysis buffer (25 mM HEPES/KOH [pH 8], 50 mM KCl, 10 mM MgSO_4_, 0.25% Triton X-100, 1 mM PMSF, 3 mM DTT, 1× complete EDTA-free protease inhibitors), supplemented with 100 μg/ml RNase A and 300 mM NaCl. Cell extracts were obtained by spinning the lysed spheroplasts at 12,000 rcf and 4°C for 5 min.

Cleared lysates (450 μl) were loaded on sucrose gradients prepared in Biocomp gradient station and sedimented in SW41 rotor (Beckman Optima L-100 XP Preparative Ultracentrifuge) at 18,000 rpm for 4 hr. Gradients were fractionated using Gilson FC203B fractionator, collecting 15 drops/fraction.

### Crosslinking Cohesin's Interfaces

Minichromosome fractions (∼100 μl) were dialyzed for 1 hr in 500 ml reaction buffer (25 mM NaPi [pH 7.4], 50 mM NaCl, 10 mM MgCl_2_, 0.25% Triton X-100) at 4°C in Slide-A-Lyzer (Pierce) columns. Samples of 95 μl were mixed with 4 μl DMSO or 5 mM bBBr freshly dissolved in DMSO and incubated for 10 min at 4°C. Reaction was quenched with 1 μl of 1 M DTT and heat denatured in 1% SDS.

### Southern Blot Analysis

Gradient fractions separated on 0.4% or 0.5% agarose gels (42 kb or 26 kb chromosomes) containing 5 μg/ml ethidium bromide at 1.1 V/cm for 44 or 60 hr at 4°C, respectively, were transferred to Immobilon-NY+ membrane (Millipore). Blots were hybridized with minichromosome (gradient sample detection) or pUC19 (genomic DNA detection) probes, scanned in FLA-7000IR (Fuji Film) and analyzed using Aida Image Analyzer v4.22.

### Western Blot Analysis

Crosslinked and denatured samples were separated in Tris-acetate gels, and blots were probed with 12CA5 α-HA antibody to detect the Smc3-TEVx3-Scc1-HA6 fusion protein.

## Supplemental Information

Document S1. Four Figures and One Table
