# Supplementary material for: Cohesin's Concatenation of Sister DNAs Maintains Their Intertwining
Source: Mol Cell. 2011 Oct 7;44(1-3):97–107. doi: 10.1016/j.molcel.2011.07.034 (PMC3240746; doi:10.1016/j.molcel.2011.07.034)
Supplement: Document S1. Four Figures and One Table [file mmc1.pdf]

Supplemental Information

Molecular Cell, *Volume 44*

Cohesin's Concatenation of Sister DNAs Maintains Their Intertwining

Ana-Maria Farcas, Pelin Uluocak, Wolfgang Helmhart, and Kim Nasmyth

Fig S1.

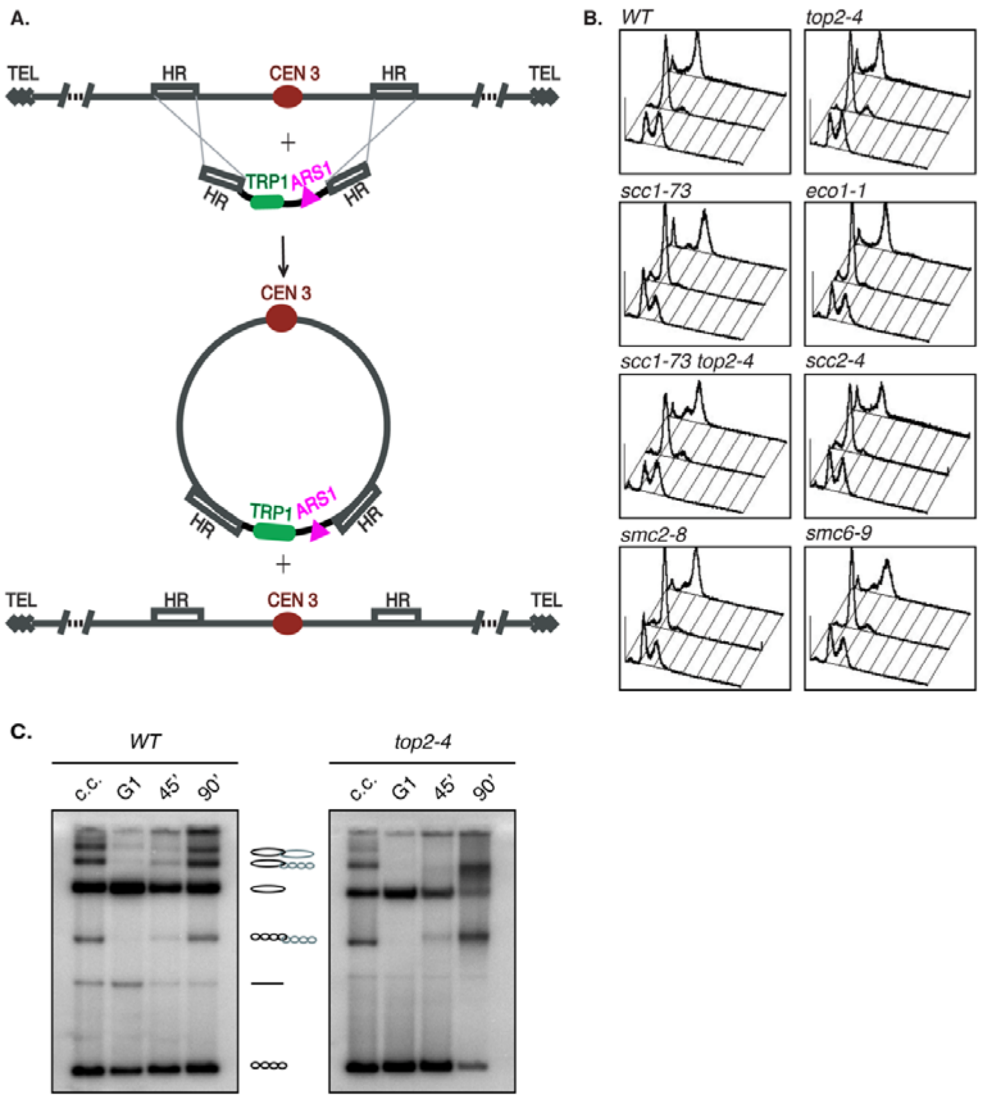

Figure S1. (A) Schematic representation of the 26 kbp circular minichromosome obtained via gap-mediated homologous recombination. (B) FACS profiles of wild type and the mutant strains (*top2-4*, *scc1-73*, *eco1-1*, *scc1-73 top2-4*, *scc2-4*, *smc2-8*, *smc6-9*) used in the experiments described in Fig. 1, Fig. 5 and Fig. 6, displaying the profiles of the cycling, G1- and metaphase- arrested cells. (C) Wild type (K16150) and *top2-4* cells (K17890) arrested with alpha-factor were released into YPD + nocodazole. Samples were collected for genomic DNA preparation and FACS.

Fig S2.

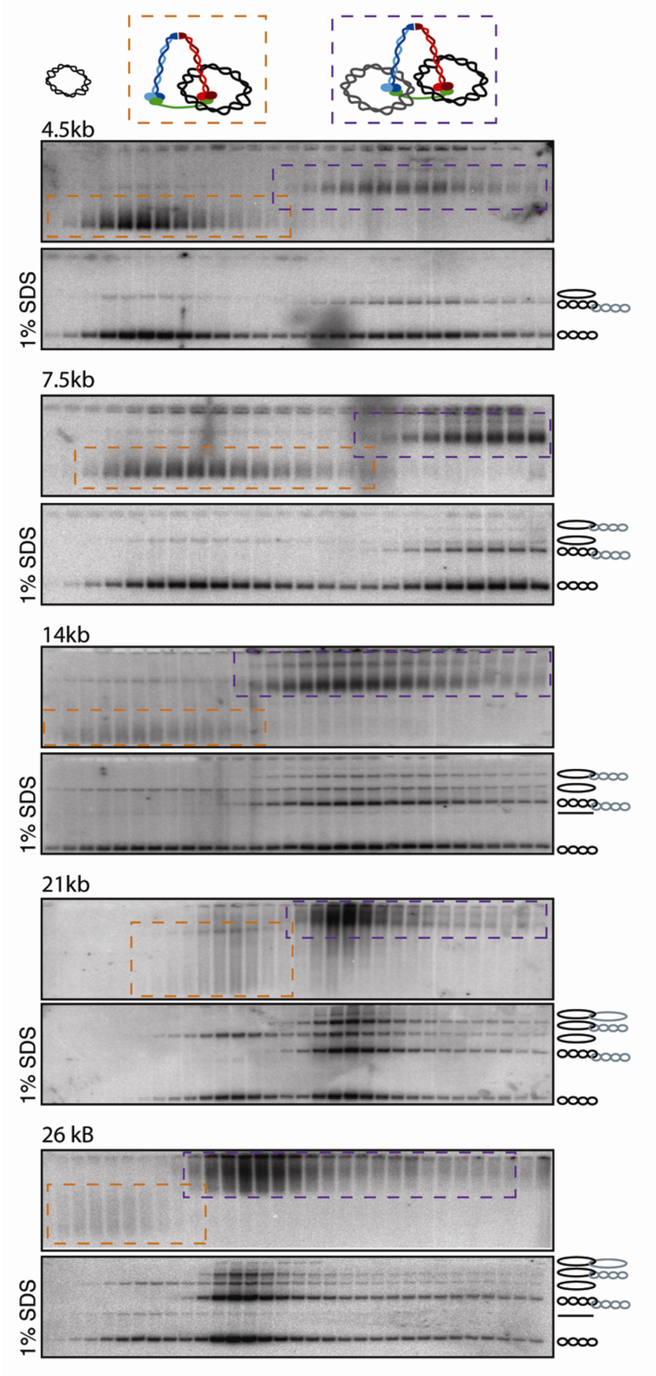

Figure S2. Measuring cohesion and catenation of circular minichromosomes of increasing size. Cleared lysates prepared from wild type cells bearing circular minichromosomes ranging from 4.5 to 26 kb (K15651, K15652, K15694, K15695 and K16150) were sedimented on sucrose gradients. Fractions were separated in agarose

gels, and monomers (dashed orange boxes) and dimers (dashed purple boxes) of circular minichromosomes were detected by Southern blotting. Sample denaturation in 1% SDS revealed that the incidence of catenated dimers is proportional to minichromosome size.

Fig S3.

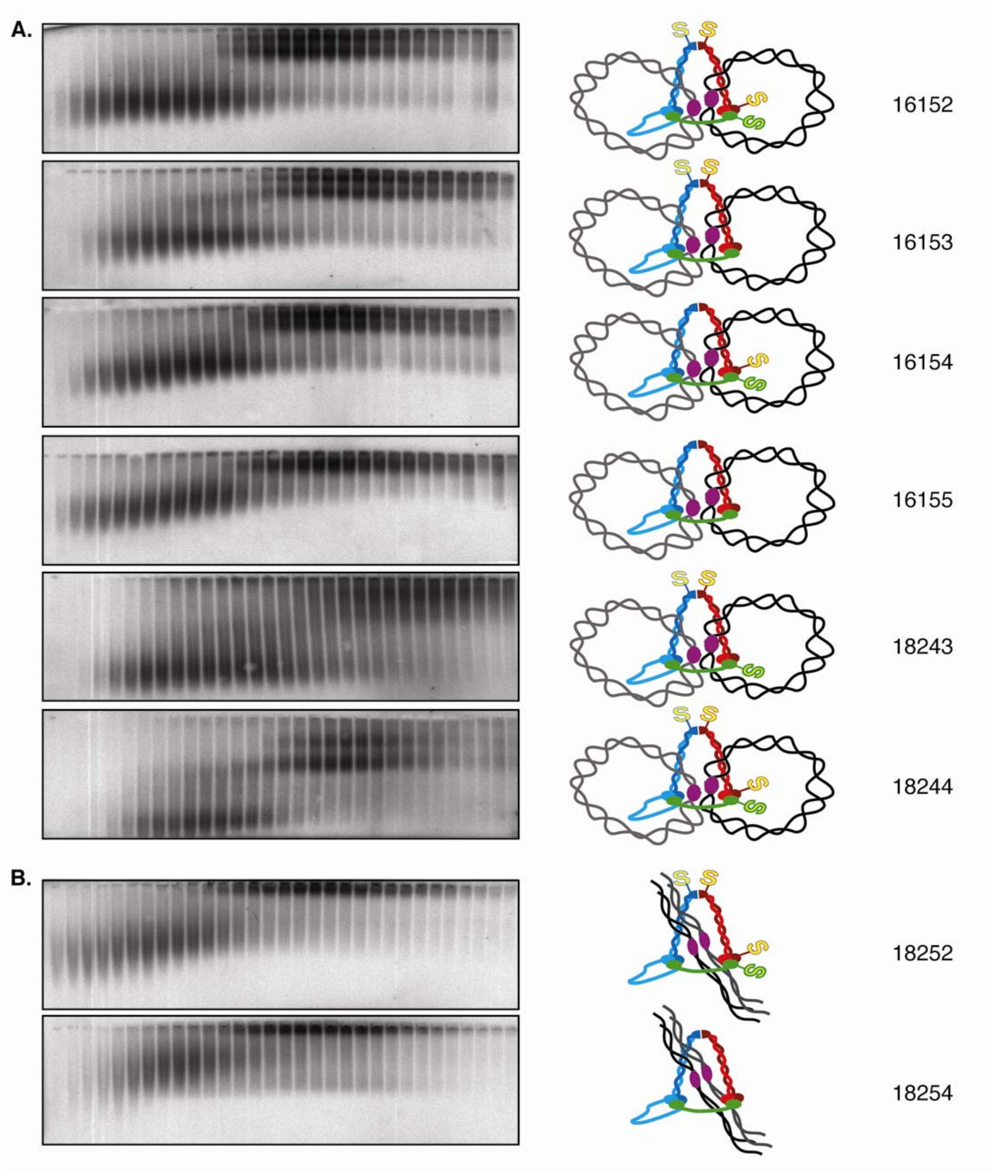

Figure S3. Preparative sucrose gradient samples for assaying the topological embrace of large circular and linear sister minichromosomes within the cohesin ring. Cleared

lysates prepared from strains containing modified versions of the cohesin complex and bearing the 26 kb circular minichromosome (A), or the 42 kb linear minichromosome (B), were sedimented in 10-45% sucrose gradients and fractions were separated in 0.5% or 0.4% agarose gels respectively.

Fig S4.

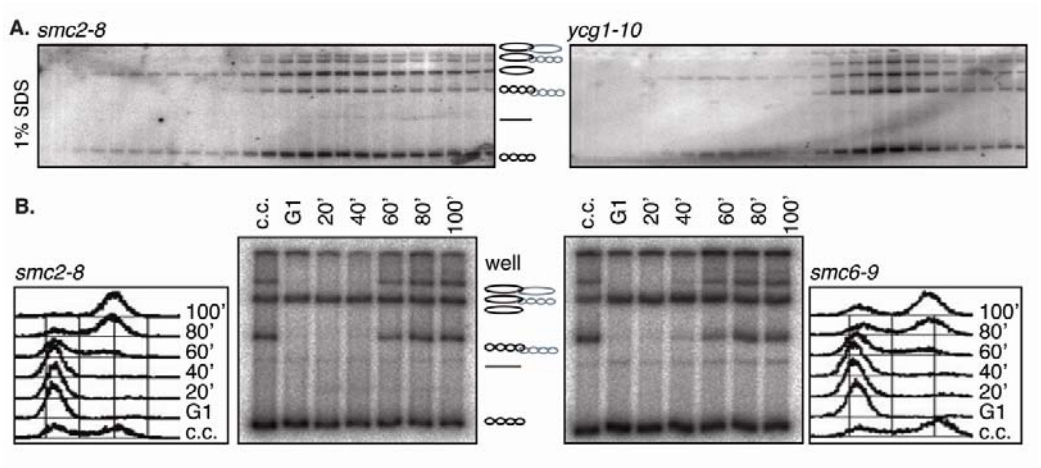

Figure S4. (A) Gradient samples extracted from *smc2-8* (K17893) and *ycg1-10* (K17894) nocodazole-arrested cells subsequent to *SMC2* and *YCG1* inactivation were heat denatured in the presence of 1% SDS. (B) Yeast strains K17893 (*smc2-8*) or K17895 (*smc6-9*) arrested with alpha-factor were released at 37°C into YPD + nocodazole. Time points were collected every 20 min for genomic DNA preparation and FACS.

Table S1: Yeast strains (W303 background)

15651	*MATa, ade2-1, trp1-1, can1-100, leu2-3,112,his3-11,15, ura3, GAL, psi+*<br>*4.5kb circular minichromosome (TRP1, ARS1, CEN1(3.3kb))*
15652	*MATa, ade2-1, trp1-1, can1-100, leu2-3,112,his3-11,15, ura3, GAL, psi+*<br>*7.5kb circular minichromosome (TRP1, ARS1, CEN1(5.5kb))*
15694	*MATa, ade2-1, trp1-1, can1-100, leu2-3,112,his3-11,15, ura3, GAL, psi+,*<br>*scc1::SCC1(TEV3)-6HA::HIS3*<br>*14.1kb circular minichromosome (SUP11, CEN3,Y', URA3)*
15695	*MATa, ade2-1, trp1-1, can1-100, leu2-3,112,his3-11,15, ura3, GAL, psi+*<br>*scc1::SCC1(TEV3)-6HA::HIS3*<br>*21.2kb circular minichromosome (RAD5, CEN4, LEU2)*
16149	*Mat a scc2-4, ade2-1, trp1-1, can1-100, leu2-3,112, his3-11,15, GAL,psi+ 3x backcrossed,*<br>*26kb circular minichromosome (TRP1, ARS1, CEN3 ~22KB ChrIII)*
16150	*Mat a, ade2-1, trp1-1, can1-100, leu2-3,112,his3-11,15, ura3, GAL, psi+*<br>*26kb circular minichromosome (TRP1, ARS1, CEN3 ~22KB ChrIII)*
16152	*Mat alpha, smc3::HIS3, scc1::KanMx, smc1::KanMx, leu2::Smc1G22CK639C-*<br>*myc9::LEU2, ura3::Scc1P-Smc3E570C-TEV3-Scc1A547C-HA6::URA3*<br>*26kb circular minichromosome (TRP1, ARS1, CEN3 ~22KB ChrIII)*
16153	*Mat a, smc3::HIS3, scc1::KanMx, smc1::KanMx, leu2::Smc1K639C-myc9::LEU2,*<br>*ura3::Scc1P-Smc3E570C-TEV3-Scc1-HA6::URA3*<br>*26kb circular minichromosome (TRP1, ARS1, CEN3 ~22KB ChrIII)*
16154	*Mat alpha, smc3::HIS3, scc1::KanMx, smc1::KanMx, leu2::Smc1G22C-myc9::LEU2,*<br>*ura3::Scc1P-Smc3-TEV3-Scc1A547C-HA6::URA3*<br>*26kb circular minichromosome (TRP1, ARS1, CEN3 ~22KB ChrIII)*
16155	*Mat a, smc3::HIS3, scc1::KanMx, smc1::KanMx, leu2::Smc1-myc9::LEU2*<br>*ura3::Scc1P-Smc3-TEV3-Scc1-HA6::URA3,*<br>*26kb circular minichromosome (TRP1, ARS1, CEN3 ~22KB ChrIII)*
17888	*Mat a, eco1-1, omns*<br>*26kb circular minichromosome (TRP1, ARS1, CEN3 ~22KB ChrIII)*
17889	*Mat a, scc1-73, omns*<br>*26kb circular minichromosome (TRP1, ARS1, CEN3 ~22KB ChrIII)*
17890	*Mat a, top2-4, omns*<br>*26kb circular minichromosome (TRP1, ARS1, CEN3 ~22KB ChrIII)*
17892	*Mat a, scc1-73 top2-4, omns*<br>*26kb circular minichromosome (TRP1, ARS1, CEN3 ~22KB ChrIII)*
17893	*Mat a, smc2-8, omns*<br>*26kb circular minichromosome (TRP1, ARS1, CEN3 ~22KB ChrIII)*
17894	*Mat a, ycg1-10, omns*<br>*26kb circular minichromosome (TRP1, ARS1, CEN3 ~22KB ChrIII)*
17895	*Mat a, smc6-9, omns*<br>*26kb circular minichromosome (TRP1, ARS1, CEN3 ~22KB ChrIII)*

18066	*Mat a, ade2-1, can1-100, his3-11,15, trp1-1, ura3-1 leu2::(GAL1-RecR::LEU2)x2 26kb circular minichromosome (TRP1, ARS1, RS-cen3-RS ~22KB ChrIII)*
18072	*Mat a, MetP-Cdc20-Leu2::cdc20, omns 26kb minichromosome (TRP1, ARS1, CEN3 ~22KB ChIII)*
18243	*Mat alpha, smc3::HIS3, scc1::KanMx, smc1::KanMx, leu2::Smc1K639C-myc9::LEU2, ura3::Scc1P-Smc3E570C-TEV3-Scc1A547C-HA6::URA3 26kb circular minichromosome (TRP1, ARS1, CEN3 ~22KB ChrIII)*
18244	*Mat a, smc3::HIS3, scc1::KanMx, smc1::KanMx, leu2::Smc1G22CK639C-myc9::LEU2, ura3::Scc1P-Smc3E570C-TEV3-Scc1A547C-HA6::URA3 26kb circular minichromosome (TRP1, ARS1, CEN3 ~22KB ChrIII)*
18249	*Mat alpha, scc1-73, omns 42kb miniChr III fragm (linear) HIS3 TRP1*
18251	*Mat a, omns 42kb miniChr III fragm (linear) HIS3 TRP1*
18252	*Mat alpha, smc3::HIS3, scc1::KanMx, smc1::KanMx, leu2::Smc1G22CK639C-myc9::LEU2, ura3::Scc1P-Smc3E570C-TEV3-Scc1A547C-HA6::URA3 42kb miniChr III fragm (linear) HIS3 TRP1*
18254	*Mat alpha, smc3::HIS3, scc1::KanMx, smc1::KanMx, leu2::Smc1-myc9::LEU2, ura3::Scc1P-Smc3-TEV3-Scc1-HA6::URA3, 42kb miniChr III fragm (linear) HIS3 TRP1*
